# Association of cognitive enhancers and incident seizure risk in dementia: a population-based study

**DOI:** 10.1186/s12877-022-03120-5

**Published:** 2022-06-03

**Authors:** Junghee Ha, Nak-Hoon Son, Young Ho Park, Eun Lee, Eosu Kim, Woo Jung Kim

**Affiliations:** 1 Department of Psychiatry, Severance Hospital, Yonsei University College of Medicine, Seoul, Republic of Korea; 2 Institute of Behavioral Science in Medicine, Yonsei University College of Medicine, Seoul, Republic of Korea; 3 Department of Statistics, Keimyung University, Daegu, Republic of Korea; 4 Department of Neurology, Seoul National University Bundang Hospital, Seoul National University College of Medicine, Seongnam, Republic of Korea; 5 Department of Psychiatry, Yongin Severance Hospital, Yonsei University College of Medicine, Yongin, Republic of Korea

**Keywords:** Cholinesterase inhibitors, Seizure, Dementia, Epilepsy, Alzheimer's disease

## Abstract

**Background:**

Although individuals with dementia have a high risk of developing seizures, whether seizures are associated with cholinesterase inhibitors, which are commonly prescribed to treat individuals with dementia, remains unknown. This study investigated the risk of incident seizure following cholinesterase inhibitor use in patients with dementia.

**Methods:**

A nationwide, nested case-control study was conducted using data from the Korean Health Insurance Review and Assessment Service (HIRA) from 2014 through 2018. A total of 13,767 participants aged 65–95 years who experienced incident seizure were propensity score-matched for medical comorbidities and drug exposure at a 1:3 ratio with a control group of 39,084 participants. The study examined the incidence of seizures in patients diagnosed with dementia within one year after receiving cognitive enhancers. Adjusted odds ratios (aORs) and 95% confidence intervals (CIs) for seizure incidence according to cholinesterase inhibitor use were analyzed using a multivariable conditional logistic regression model.

**Results:**

There was no statistically significant association between duration of cholinesterase inhibitors use and seizure risk. Although there was slight increased seizure risk in patient after receiving donepezil for 1 year compared to memantine, subgroup analyses stratified age and sex did not reveal any significant association between cholinesterase inhibitors use and late-onset seizure.

**Conclusions:**

Our findings suggest no immediate increase in seizure risk is associated with cholinesterase inhibitor use, although the risk of seizure in patients with dementia did increase after one year of continued medication intake. Further study is required to obtain confirmatory results on the seizure-related safety of cognitive enhancers in patients with dementia.

**Supplementary Information:**

The online version contains supplementary material available at 10.1186/s12877-022-03120-5.

## Introduction

Dementia is a broad-spectrum disease characterized by neurological abnormalities and disturbances in various behaviors and psychological functions. Comorbidity between dementia and seizure is more common than expected [1, 2], and patients with Alzheimer’s disease (AD) have a two- to six-fold increased risk of developing seizure and epilepsy compared with healthy individuals [3]. Although seizures were previously known to occur primarily in the late stages of dementia, they have also been observed in the prodromal or early stages [4, 5]. Growing evidence suggests that the neuronal hyperexcitability that occurs in seizures may contribute to neuropathological burden and that prolonged or recurring seizures may cause or exacerbate cognitive impairment [6–8].

The WHO Adverse Drug Reaction database has reported that cholinesterase inhibitors, often prescribed to patients with AD and related dementias, may lower the seizure threshold and provoke seizures [9]. However, the findings have been inconsistent. In a small, randomized, double-blinded, controlled trial, donepezil was not associated with increased seizure frequency in patients with epilepsy and memory complaints [10], whereas another open-label study demonstrated a tendency for increased seizure in individuals taking donepezil [11]. Memantine, a noncompetitive N-methyl-Daspartate receptor antagonist, has also demonstrated to have both pro- and anticonvulsant effects in animal models [12], but previous human studies have shown that memantine has a relatively lower seizure risk than donepezil [9]. In particular, a clinical study showed that administration of memantine to patients with seizures not only improved cognition but also reduced the frequency of seizures [13].

Surprisingly few systematic studies have addressed seizure risk in the older individuals taking cholinesterase inhibitors. Previous studies examining whether cholinesterase inhibitors induce seizures have failed to consider confounding medications and had limited sample sizes [10, 14, 15]. The association between seizures and cholinesterase inhibitor use remains unclear. Although monoclonal antibodies direct against amyloid for the treatment of AD have been recently introduced, their effectiveness and safety have not been sufficiently verified, and it is estimated that the conventional cognitive enhancers such as cholinesterase inhibitors will be widely used as they are now. Therefore, studies on the safety of cognitive enhancers in patients with dementia have sufficient clinical significance. In this study, we investigated whether the use of cholinesterase inhibitors lead to increased risk of seizure compared to memantine and examined the effects of drug types and treatment duration on seizure threshold, adjusting for comorbidity and concomitant exposure to other drugs in a large, population-based cohort in South Korea.

## Methods

### Data sources

We performed a retrospective analysis of a large, population-based database from the Korean Health Insurance and Review Assessment (HIRA), which includes all health insurance claims from the entire population of South Korea. The HIRA research database contains information on patients’ demographics; diagnostic codes by the International Classification of Diseases, 10th Revision (ICD-10); and details of medical examinations and treatments, such as tests, procedures, and drug prescriptions [16]. This study was approved by the institutional review board of the university affiliated Hospital (No. 9–2020-0001) with a waiver of informed consent.

### Study design and population

**Fig. 1:**
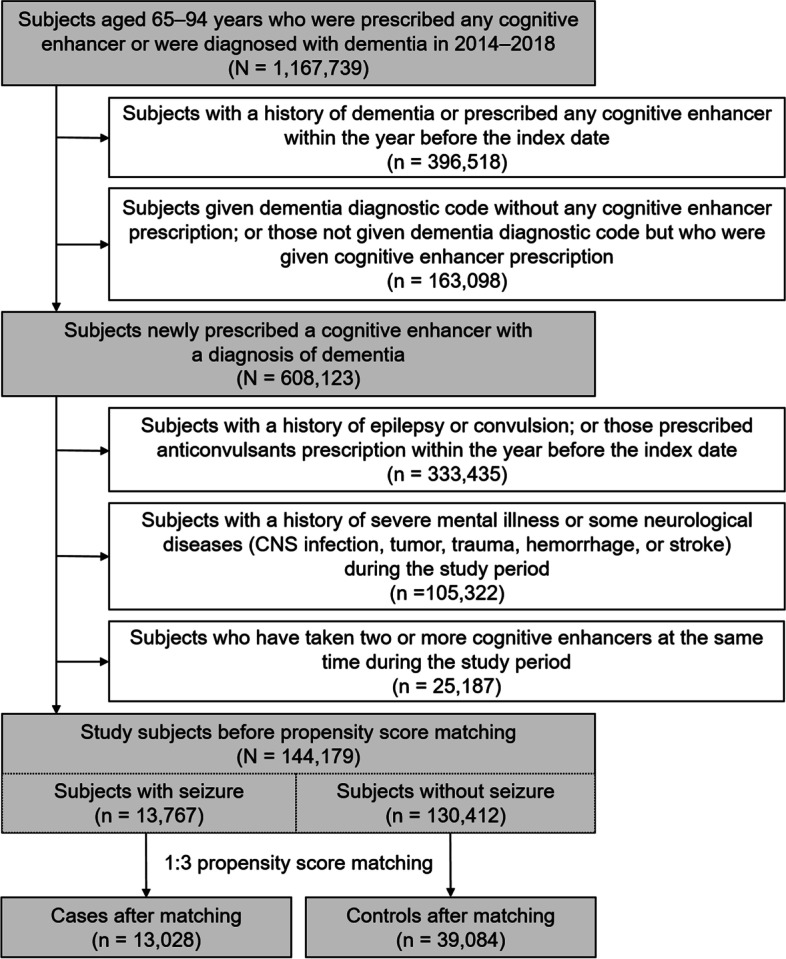
Flowchart of the study participant selection process

This nested case-control study used the HIRA claims database. Figure 1 shows the flow of our sample selection process. We first extracted a cohort of 1,167,739 patients aged 65–94 years who were prescribed cognitive enhancers (donepezil, rivastigmine, galantamine, or memantine) or diagnosed with dementia (Alzheimer’s dementia [F00.X, G30.X] or vascular dementia [F01.X]) between January 1, 2014, and December 31, 2018.

The index date was defined as either the first diagnosis of dementia or the first prescription of any cognitive enhancer following a washout period of one year, whichever was earlier. Subjects with a history of dementia and those who were prescribed any cognitive enhancer during the year before the index date were excluded (*n* = 396,518). To ensure diagnostic validity, we confined the study sample to those for whom diagnosis and medication use coincided. We thus excluded a) subjects with a dementia diagnostic code but no cognitive enhancer prescription and b) those with a cognitive enhancer prescription but no dementia diagnostic code during the study period (*n* = 163,098).

In this study, we set a group with newly developed seizures and a group without seizures as controls after dementia diagnosis and investigated whether cholinesterase exposure is associated with the risk of seizures. We also considered a washout period for the outcome (seizure), excluding 333,435 subjects with a history of epilepsy (G40.X, G41.X) or convulsion (R56.8) and those who received prescriptions for anticonvulsants in the year before the index date (Supplementary File, S1).

During the study period, subjects with a history of severe mental illness or certain neurological diseases (i.e., central nervous system [CNS] infection, CNS tumor, head trauma, cerebral hemorrhage, and stroke) (*n* = 105,322) and subjects who had taken two or more cognitive enhancers at the same time (*n* = 25,187) were excluded (Fig. 1).

### Case and control definitions

The study outcome was the presence of seizure, which was defined as having received an anticonvulsant prescription within 30 days after the registration of a diagnostic code of epilepsy or convulsion. We defined the case group as subjects having new-onset seizures within the study period. All other subjects (i.e., those without any seizure event in the study period) were defined as the control group. In total, we included 144,179 study subjects: 13,767 cases and 130,412 controls.

Each case was propensity score-matched with three controls based on the following variables: related comorbidities and comedication that were significantly different between the two groups. Comorbidities included cerebrovascular disease, diabetes with chronic complications, sleep disorders, hemi-/paraplegia, substance issues, Parkinsonism, renal failure, moderate/severe liver disease, and chronic pulmonary disease; comedications included analgesics, antibacterials, anticholinergics, antidepressants, antifungals, antihistamines, anti-Parkinsonian drugs, antipsychotics, antituberculosis drugs, benzodiazepines, glucocorticoids, and psychostimulants. The numbers of cases and controls in the matched sample were 13,028 and 39,084, respectively.

### Exposures

Among four cognitive enhancers, we postulated that cholinesterase inhibitors (i.e., donepezil, rivastigmine, and galantamine) would have more proconvulsive effects than memantine [9]. The adjusted odds ratios (aORs) for each cholinesterase inhibitor to induce seizure relative to a reference group (memantine users) were estimated using logistic regression. We examined the aORs after one year of treatment in the primary analyses. To test the robustness of the results of these analyses, we conducted follow-up sensitivity analyses using different time windows (i.e., 1, 3, and 6 months) for each case and control group.

### Confounders

Various confounders might impact the association between the use of cognitive enhancers and the risk of seizure. The comorbidities and comedications investigated in this study were determined based on previous studies [17–20]. Age, sex, residential area, and type of dementia were assessed on the index date, whereas comorbidities (cerebrovascular disease, diabetes with chronic complication, sleep disorders, hemi-/paraplegia, substance issues, Parkinsonism, renal failure, moderate/severe liver disease, and chronic pulmonary disease) and comedications (analgesics, antibacterials, anticholinergics, antidepressants, antifungals, antihistamines, anti-parkinsonian drugs, antipsychotics, antituberculosis drugs, benzodiazepines, glucocorticoids, and psychostimulants) were assessed throughout the study period. Subgroup analyses were performed for age (< 80 years vs. ≥ 80 years) and sex (male vs. female).

### Statistical analysis

Categorical variables were summarized using frequencies and percentages and compared using the Chi-square test or Fisher’s exact test. Continuous variables were summarized using means and standard deviations (SDs) and compared using the two-sample t-test. The Shapiro–Wilk test was used to test the normality of the distribution. All statistical tests were two-sided, and *p*-values < 0.05 were considered statistically significant. Data matching was conducted to adjust for differences between subjects across multiple variables presented previously [21]. The matching ratio was 1:3 (case: control). Each parameter was evaluated using logistic regression in the LOGISTIC procedure of SAS (Version 9.4; SAS Institute, Cary, NC), using observed data from each patient with no imputation for missing data.

## Results

**Fig. 2:**
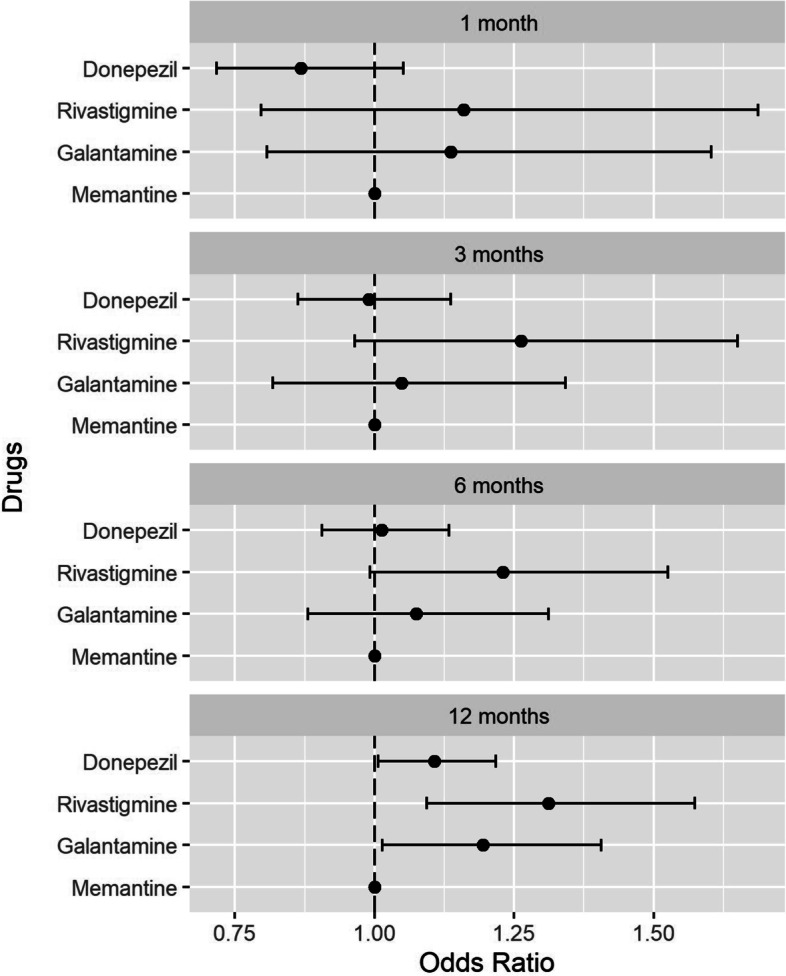
Sensitivity analyses for risk of seizures according to duration of drug administration. Adjusted odds ratios with 95% confidence intervals for multivariable models to evaluate associations between cholinesterase inhibitor use and incident seizure within 12 months. Analysis was adjusted for the following covariates: comorbidities (cerebrovascular disease, diabetes with chronic complications, sleep disorders, hemi-/paraplegia, substance issues, Parkinsonism, renal failure, liver disease, and chronic pulmonary disease) and comedications (analgesics, antibacterials, anticholinergics, antidepressants, antifungals, antihistamines, anti-parkinsonian drugs, antipsychotics, antituberculosis drugs, benzodiazepines, glucocorticoids, and psychostimulants)

We identified 1,167,739 patients who received a dementia diagnosis during 2014–2018 and included 144,179 subjects with newly diagnosed dementia and without a history of seizure who were taking one cognitive enhancer, as presented in Fig. 1. Before case-control matching was performed, the mean (± *SD*) follow-up durations for users of each cognitive enhancer were as follows: 475.77 (± 460.94) days for donepezil users, 446.74 (± 476.33) days for rivastigmine users, 446.58 (± 464.11) days for galantamine users, and 411.85 (± 446.98) days for memantine users. A total of 13,767 participants aged 65–95 years with seizures were matched for medical comorbidities and concomitant medications, with 39,084 participants included as a control group. The characteristics of the 13,028 cases with seizures and the 39,084 controls without seizures are given in Table 1. After matching, no statistically significant differences in medical comorbidities or concomitant medications were identified between the case and control groups. A conditional logistic model was used to evaluate the association of seizures with cognitive enhancer treatment. Table 2 demonstrates that one year of cholinesterase inhibitor use was associated with an increased risk of seizure compared with memantine use. The risk of seizure varied across different classes of cholinesterase inhibitors. We found that seizure risk was highest for rivastigmine (aOR = 1.31, 95% CI: 1.09–1.57), followed by galantamine (aOR = 1.19, 95% CI: 1.01–1.41), and donepezil (aOR = 1.11, 95% CI: 1.01–1.22). Further sensitivity analyses using different time windows for drug administration (1, 3, and 6 months) did not identify a statistically significant association between these drugs and seizure risk, and seizure incidence did not increase according to the duration of drug administration (aORs [CI] for donepezil use < 1, 3, 6, and 12 months compared with memantine use were 0.87 [0.72–1.05], 0.99 [0.86–1.14], 1.01 [0.91–1.13], and 1.11 [1.01–1.22], respectively) (Fig. 2). Statistically significant associations were not maintained in subgroup analyses stratified by age (< 80 years vs. ≥ 80 years) or sex (male vs. female) (Supplementary File, S2-3).

**Table 1:** Baseline characteristics of the study subjects

	Before PSM (N = 144,179)					After PSM (*N* = 52,184)				
	No seizure (n = 130,412)		Seizure (n = 13,767)		*p*	No seizure (*n* = 39,084)		Seizure (*n* = 13,028)		*p*
Age group, *n* (%)					<.0001					<.0001
65–70 years	6,128	(4.7)	999	(7.3)		2,154	(5.5)	928	(7.1)	
70–74 years	12,269	(9.4)	1,884	(13.7)		4,190	(10.7)	1,746	(13.4)	
75–79 years	25,838	(19.8)	3,531	(25.7)		8,519	(21.8)	3,315	(25.5)	
80–84 years	35,290	(27.1)	3,762	(27.3)		11,106	(28.4)	3,564	(27.4)	
85–89 years	31,634	(24.3)	2,529	(18.4)		8,641	(22.1)	2,432	(18.7)	
90–94 years	19,253	(14.8)	1,062	(7.7)		4,474	(11.5)	1,043	(8.0)	
Sex, *n* (%)					<.0001					<.0001
Male	37,301	(28.6)	4,500	(32.7)		11,406	(29.2)	4,194	(32.2)	
Female	93,111	(71.4)	9,267	(67.3)		27,678	(70.8)	8,834	(67.8)	
Residential area, *n* (%)					0.447					0.136
Metropolitan area	46,729	(35.8)	4,978	(36.2)		13,803	(35.3)	4,695	(36.0)	
Non-metropolitan area	83,683	(64.2)	8,789	(63.8)		25,281	(64.7)	8,333	(64.0)	
Type of dementia, *n* (%)					0.008					0.700
Alzheimer’s dementia	125,141	(96.0)	13,146	(95.5)		37,399	(95.7)	12,456	(95.6)	
Vascular dementia	5,271	(4.0)	621	(4.5)		1,685	(4.3)	572	(4.4)	
Donepezil use, *n* (%)					0.173					0.834
Yes	119,138	(91.4)	12,624	(91.7)		35,782	(91.6)	11,935	(91.6)	
No	11,274	(8.6)	1,143	(8.3)		3,302	(8.5)	1,093	(8.4)	
Rivastigmine use, *n* (%)					<.0001					0.032
Yes	1,459	(1.1)	234	(1.7)		549	(1.4)	217	(1.7)	
No	128,953	(98.9)	13,533	(98.3)		38,535	(98.6)	12,811	(98.3)	
Galantamine use, *n* (%)					0.016					0.265
Yes	2,421	(1.9)	296	(2.2)		798	(2.0)	287	(2.2)	
No	127,991	(98.1)	13,471	(97.9)		38,286	(98.0)	12,741	(97.8)	
Memantine use, *n* (%)					<.0001					0.027
Yes	7,394	(5.7)	613	(4.5)		1,955	(5.0)	589	(4.5)	
No	123,018	(94.3)	13,154	(95.6)		37,194	(95.0)	12,452	(95.5)	
Comorbidities, *n* (%)										
Cerebrovascular disease	43,003	(33.0)	6,761	(49.1)	<.0001	18,320	(46.9)	6,103	(46.9)	0.956
Diabetes with chronic complication	22,078	(16.9)	3,264	(23.7)	<.0001	8,886	(22.7)	2,960	(22.7)	0.971
Sleep disorders	5,349	(4.1)	1,252	(9.1)	<.0001	2,865	(7.3)	998	(7.7)	0.213
Hemi-/paraplegia	4,731	(3.6)	1,087	(7.9)	<.0001	2,272	(5.8)	766	(5.9)	0.779
Substance issues	3,405	(2.6)	560	(4.1)	<.0001	1,356	(3.5)	479	(3.7)	0.266
Parkinsonism	2,175	(1.7)	584	(4.2)	<.0001	1,105	(2.8)	429	(3.3)	0.007
Renal failure	1,836	(1.4)	399	(2.9)	<.0001	991	(2.5)	311	(2.4)	0.347
Moderate/severe liver disease	1,575	(1.2)	223	(1.6)	<.0001	585	(1.5)	211	(1.6)	0.322
Chronic pulmonary disease	612	(0.5)	86	(0.6)	0.013	221	(0.6)	78	(0.6)	0.663
Comedications, *n* (%)										
Analgesics	117,460	(90.1)	13,256	(96.3)	<.0001	37,666	(96.4)	12,520	(96.1)	0.155
Antibacterials	121,409	(93.1)	13,516	(98.2)	<.0001	38,394	(98.2)	12,778	(98.1)	0.254
Anticholinergics	62,559	(48.0)	8,458	(61.4)	<.0001	23,560	(60.3)	7,867	(60.4)	0.832
Antidepressants	55,035	(42.2)	8,991	(65.3)	<.0001	24,913	(63.7)	8,275	(63.5)	0.644
Antifungals	29,600	(22.7)	4,111	(29.9)	<.0001	11,182	(28.6)	3,739	(28.7)	0.845
Antihistamines	115,300	(88.4)	13,075	(95.0)	<.0001	37,102	(94.9)	12,346	(94.8)	0.462
Anti-Parkinsonian drugs	7,608	(5.8)	1,919	(13.9)	<.0001	4,255	(10.9)	1,477	(11.3)	0.155
Antipsychotics	35,117	(26.9)	5,748	(41.8)	<.0001	15,319	(39.2)	5,108	(39.2)	0.979
Antituberculosis drugs	2,399	(1.8)	300	(2.2)	0.005	857	(2.2)	279	(2.1)	0.729
Benzodiazepines	93,000	(71.3)	12,432	(90.3)	<.0001	35,106	(89.8)	11,694	(89.8)	0.841
Glucocorticoids	71,133	(54.5)	9,343	(67.9)	<.0001	26,251	(67.2)	8,718	(66.9)	0.602
Psychostimulants	505	(0.4)	141	(1.0)	<.0001	221	(0.6)	88	(0.7)	0.157

*PSM* Propensity Score Matching

**Table 2:** One-year risk of seizures in users of each cholinesterase inhibitor compared with memantine users

	Before PSM (N = 144,179)			After PSM (N = 52,184)		
	OR	(95% CIs)	*p*	OR	(95% CIs)	*p*
Memantine	1.000	(Reference)		1.000	(Reference)	
Donepezil	1.061	(0.973–1.158)	0.181	1.107	(1.007–1.217)	0.035
Rivastigmine	1.135	(0.960–1.343)	0.137	1.312	(1.093–1.574)	0.004
Galantamine	1.087	(0.935–1.264)	0.277	1.194	(1.014–1.406)	0.034

*PSM* Propensity Score Matching, *OR* Odds Ratio, *CI* Confidence Interval

## Discussion

In this study, we did not observe an immediate increase in the risk of seizure with cholinesterase inhibitor use; however, an increased risk of seizure was identified in patients with dementia after one year of cholinesterase inhibitor administration. To the best of our knowledge, this is the first large, population-based study to investigate the association between cognitive enhancers and seizures in patients with dementia. Although innovative AD drugs such as amyloid beta-directed monoclonal antibody have recently been introduced, further studies should be conducted to confirm the efficacy and safety of the drugs. For the time being, it is estimated that conventional cognitive enhancers such as cholinesterase inhibitors will be widely used as they are in the present, hence our study on the safety of cholinesterase inhibitors will provide valuable information in real-world practice.

The overall results of this population-based case-control study indicate that the association between cholinesterase inhibitors and seizure in patients with dementia was not significant. Although a significant association was observed between seizure incidence and one year of cholinesterase inhibitor use, this association was not maintained in patients with shorter durations of use or in subgroup analyses. Neither sensitivity analysis nor further stratification by age or sex revealed statistically significant associations between cholinesterase inhibitor use and seizure incidence. It is unclear whether this increased risk at one year was the result of the cumulative drug effect of cholinesterase inhibitors compared to memantine. Rather, the increased risk of seizures may have been due to the degenerative changes resulting from dementia progression, as previous studies have shown that seizures are correlated with dementia severity [22, 23]. Further studies considering clinical variables including dementia severity is required to confirm the long-term safety of cognitive enhancers in relation to seizure risk in dementia patients.

Lower seizure thresholds have been noted with cholinergic agent use in animal models [24], although the mechanism of convulsive action associated with cholinesterase inhibitors is not fully understood. Gradual loss of hippocampal cholinergic neurotransmission―causing progressive deterioration of memory results in AD [25] ―and a similar depletion in hippocampal acetylcholine levels have been implicated as causal factors in an experimental model of epilepsy [26]. Donepezil, the most commonly prescribed cholinesterase inhibitor, reversibly inactivates cholinesterase, inhibiting the hydrolysis of acetylcholine and increasing its concentration in the extrasynaptic space of cholinergic neurons. Reductions in cortical dopamine and serotonin levels have also been observed in previous animal studies with cholinesterase inhibitors, which may lead to decreased seizure thresholds, as reduced monoamine levels are related to increased seizure risk [27]. On the other hand, the prolonged presence of acetylcholine in the hippocampus and cortex induces sustained synaptic enhancement in CA1 pyramidal neurons, which may contribute to memory improvement [28].

Few studies have investigated the association between cognitive enhancers and seizures. One patient with mild AD who took 10 mg donepezil daily for 3 weeks experienced seizures during the treatment period, and the seizures recurred when donepezil was re-administered [29]. Another case report found that metabolic disturbances induced by cholinesterase inhibitors may lead to hyponatraemic seizures [30]. However, the subject described in the case report had alcohol dependency and was prescribed both donepezil and memantine, preventing these results from being easily generalized. Treatment with cholinesterase inhibitors has provoked seizures in patients with epilepsy [11], and other centrally acting cholinesterase inhibitors, such as tacrine, velnacrine, and physostigmine, might induce convulsions in patients with AD [31, 32]. However, a randomized, double-blinded study of patients with epilepsy and memory complaints failed to demonstrate increased seizure frequency in the group treated with donepezil [10]. Furthermore, an animal experiment found that donepezil treatment after seizures induced protective effects, reducing neuronal degeneration, oxidative damage, and microglial activation [33]. The study for licensing donepezil did not consider seizure as a serious adverse event [34]. Similarly, in a British observational cohort study of 1,762 patients, convulsion or seizure was listed as a rare event in donepezil treatment: 10 cases were noted, only two of which were considered to be causally related to treatment according to pragmatic classification [14].

Whether cholinesterase inhibitors contribute to seizure development in older adult patients remains unclear. Previous studies have been limited by small sample sizes and insufficient consideration of concurrent medication use. Given the high incidence of polypharmacy among older adults, concomitant drug administration that raises the seizure threshold could be a crucial contributor to seizure development. Therefore, we conducted this nested case-control study from a nationwide cohort to include a larger number of patients with dementia with reliable data for prescribed cholinesterase inhibitors and comedication.

We compared the seizure risk associated with several cognitive enhancers, using memantine as the reference group because fewer seizure risks have been reported for this cholinesterase inhibitor [9]. To maintain homogeneity within the group, we included only patients with dementia who were prescribed cognitive enhancers and excluded those who were not on medication after a diagnosis of dementia, assuming that they had either mild cognitive impairment or were misdiagnosed.

Our findings revealed no serious detrimental role for cholinesterase inhibitors in seizure development, consistent with the aforementioned study results [10, 14]. The null association between cholinesterase inhibitor use and seizure risk may be attributed to the heterogeneity of dementia characteristics. Dementia is a multifactorial disease resulting from a combination of ageing, genetic predisposition, and exposure to various circumstances, such as head trauma, viruses, and toxins [35]. The frequency of seizures gradually increases as the disease progresses, which has been reaffirmed by the results of this study [23]. However, to clarify the association between cholinesterase inhibitors and seizure in patients with dementia, adequately powered, randomized controlled trials or larger longitudinal studies with substantial follow-up periods are needed.

This study has several strengths. The analyses used data from a large, nationwide, longitudinal cohort and considered many covariates, allowing for adjustment for possible confounding factors. Most importantly, to the best of our knowledge, this is the first study to examine the association between cognitive enhancers and seizure development using validated, nationwide, longitudinal cohort data. This study also has several limitations. First, it uses administrative databases, making it susceptible to errors arising from coding inaccuracies. However, in previous studies comparing the diagnosis in this claim database with the actual diagnosis in hospital records, the positive predictive value for diagnostic accuracy was 83.4% for ischemic stroke [36] and 83% for Alzheimer's disease [37]. We also applied a definition for seizure that has been validated in previous studies [38, 39]. However, careful interpretation is needed, as it is a claim-based administrative data, it may not reflect actual medication compliance. Second, dose dependency was not evaluated in this study. We sought to reduce this limitation by conducting sensitivity analyses using different treatment duration time points to eliminate differences in exposure**.** Third, although we used propensity score matching to reduce potential selection bias in identifying cases or control, and adjusted concomitant medication and comorbidities, there are still unmeasured confounding factors that could have influenced the results. Finally, insufficient information was available regarding the severity of the patients’ dementia, which may affect seizure threshold. Additional large prospective studies that include these clinical variables are needed to confirm the long-term safety of cognitive enhancers with regard to seizure risk in dementia patients.

## Conclusions

An increased risk of seizure was found in patients who took cholinesterase inhibitors for one year; however, statistically significant associations were not maintained when the patients were divided into subgroups, and no associations with seizure risk were observed in patients when examined for shorter treatment durations. These results may suggest that seizure is more likely to result from degenerative changes due to dementia progression than to the effects of medication. Additional prospective controlled trials, especially trials that consider dementia severity, are required to confirm the observed null association.

## Supplementary Information

**Additional file 1. **Supplementary File.

## Data Availability

The data that the findings of this study are HIRA research data (M20200302349) and are stored on a separate server managed by the HIRA. The datasets generated and analyzed during the current study are not publicly available due to HIRA restrictions. Access to the data is regulated by Korean law and the Korean National Institute for Health and Welfare. Interested parties may submit an application to the HIRA for access. The HIRA accepts applications via their website (https://opendata.hira.or.kr/) and requires a study proposal as well as ethics approval from the researcher’s institutional review board.

## Abbreviations

AOR: Adjusted odds ratio
CI: Confidence interval
PSM: Propensity score matching
AD: Alzheimer's disease
