# Supplementary material for: Association of cognitive enhancers and incident seizure risk in dementia: a population-based study
Source: BMC Geriatr. 2022 Jun 3;22:480. doi: 10.1186/s12877-022-03120-5 (PMC9166339; doi:10.1186/s12877-022-03120-5)

Supplementary File

Figure S1. Study design

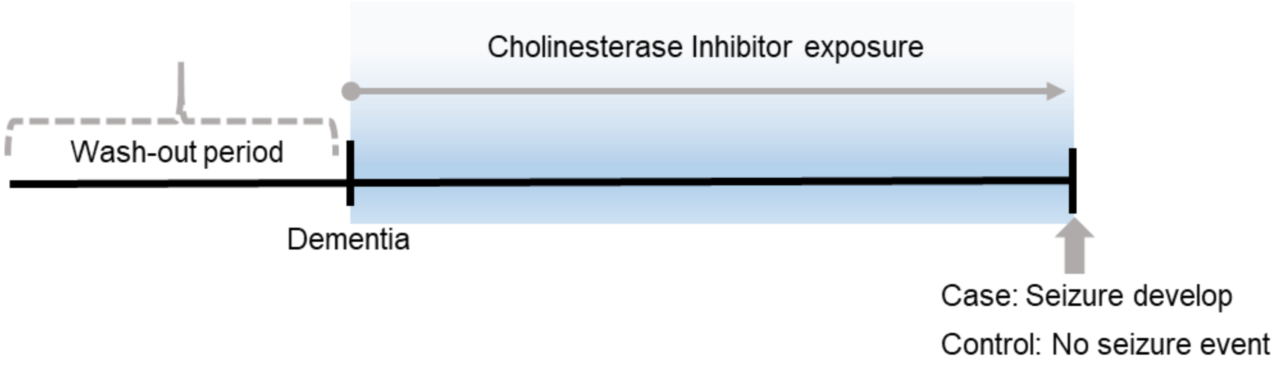

Figure S2. Association between cholinesterase inhibitor use and seizure according to age.

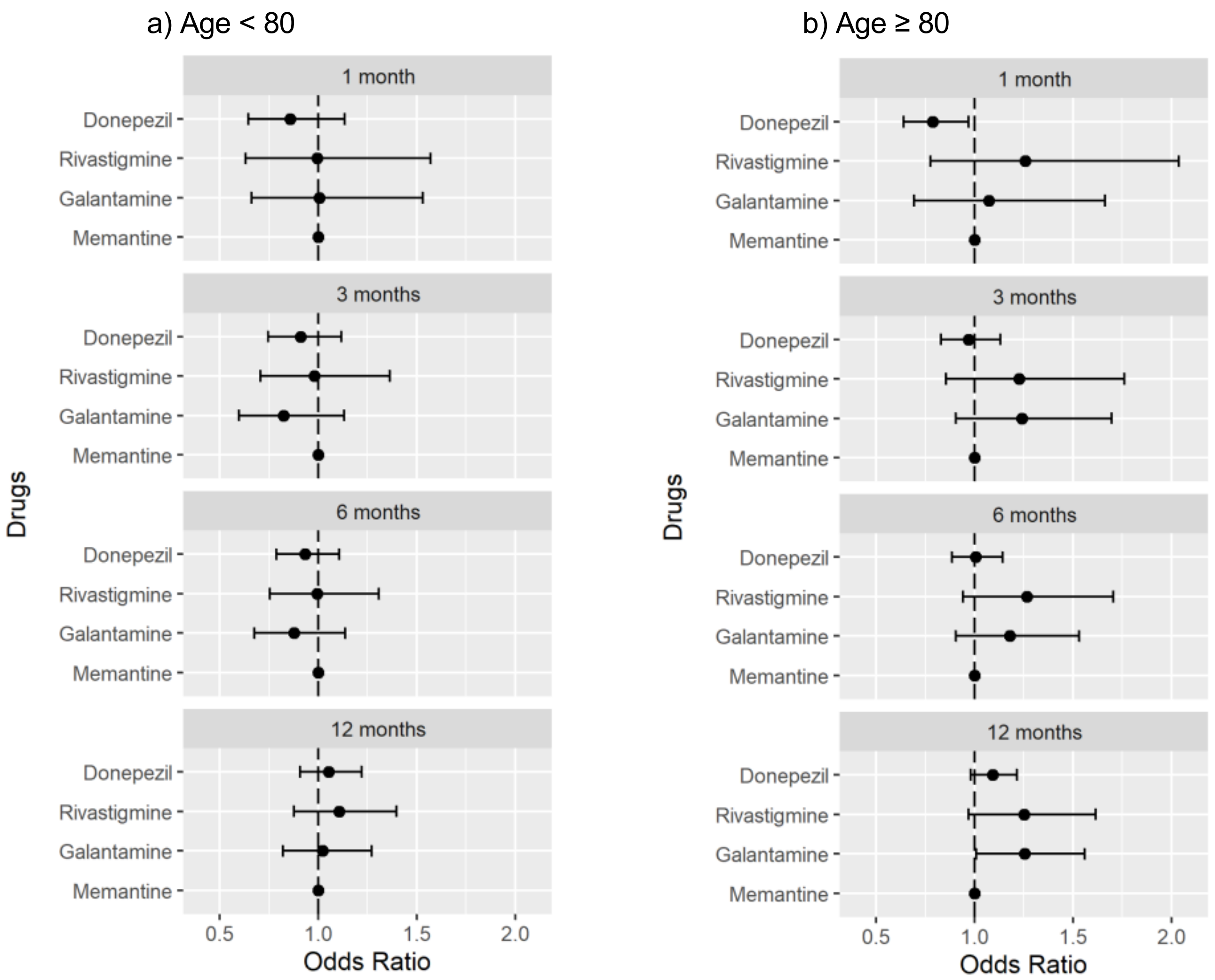

Figure S3. Association between cholinesterase inhibitor use and seizure according to sex.

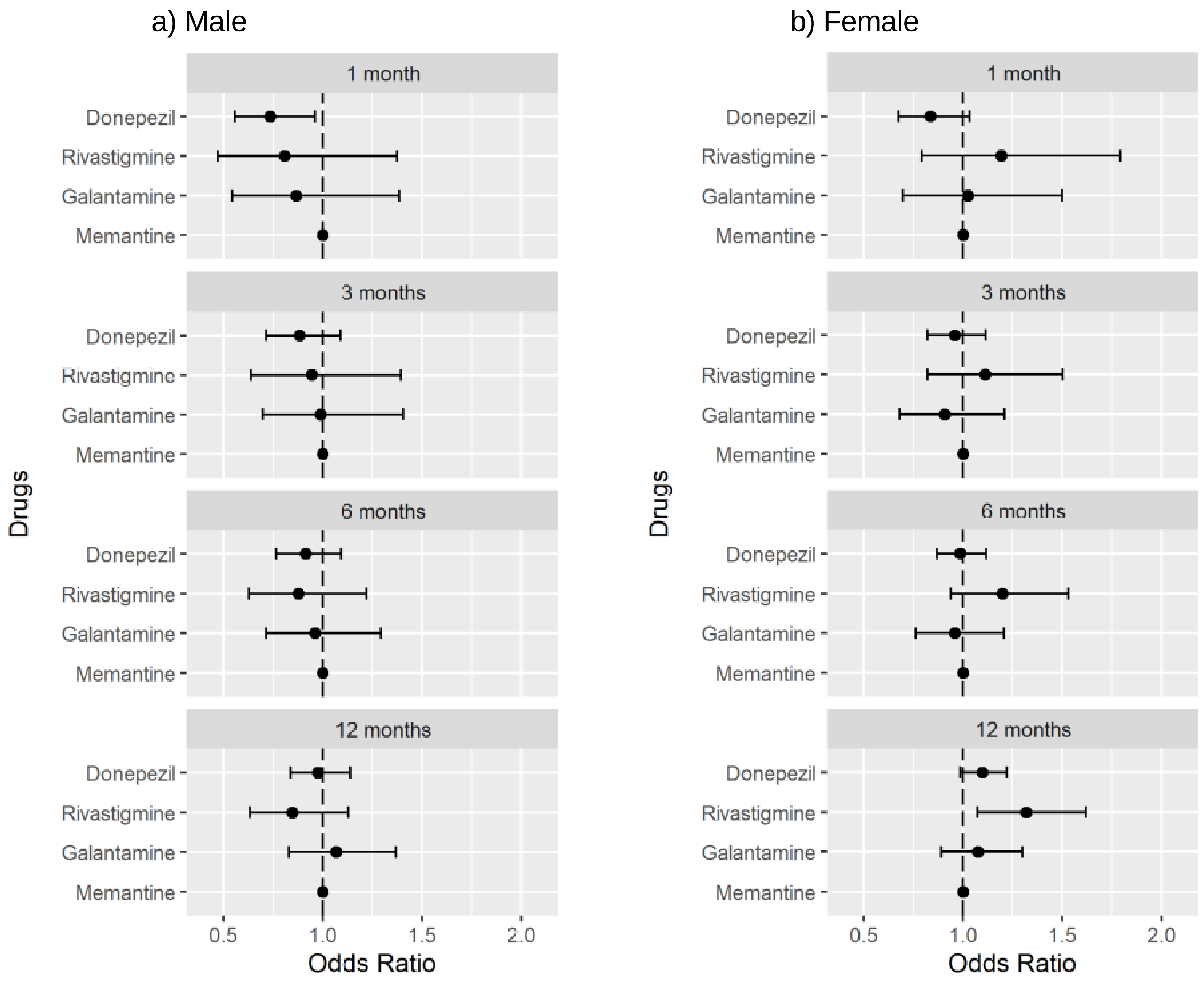

Supplement: Supplementary file 1 — Additional file 1. Supplementary File. [file 12877_2022_3120_MOESM1_ESM.pdf]
